# Classification of Ankle Joint Stiffness during Walking to Determine the Use of Ankle Foot Orthosis after Stroke

**DOI:** 10.3390/brainsci11111512

**Published:** 2021-11-15

**Authors:** Yusuke Sekiguchi, Keita Honda, Dai Owaki, Shin-Ichi Izumi

**Affiliations:** 1 Department of Physical Medicine and Rehabilitation, Tohoku University Graduate School of Medicine, 2-1, Aoba-ku, Seiryo-machi, Sendai 980-8575, Japan; keita.honda.d2@tohoku.ac.jp (K.H.); izumis@med.tohoku.ac.jp (S.-I.I.); 2 Department of Robotics, Graduate School of Engineering, Tohoku University, 6-6-01 Aoba, Aramaki, Aoba-ku, Sendai 980-8579, Japan; owaki@tohoku.ac.jp; 3 Graduate School of Biomedical Engineering, Tohoku University, 2-1, Seiryo-machi, Sendai 980-8575, Japan

**Keywords:** categorization, gait, stroke, quasi-joint stiffness, ankle joint, orthosis

## Abstract

Categorization based on quasi-joint stiffness (QJS) may help clinicians select appropriate ankle foot orthoses (AFOs). The objectives of the present study were to classify the gait pattern based on ankle joint stiffness, also called QJS, of the gait in patients after stroke and to clarify differences in the type of AFO among 72 patients after stroke. Hierarchical cluster analysis was used to classify gait patterns based on QJS at least one month before the study, which revealed three distinct subgroups (SGs 1, 2, and 3). The proportion of use of AFOs, articulated AFOs, and non-articulated AFOs were significantly different among SGs 1–3. In SG1, with a higher QJS in the early and middle stance, the proportion of the patients using articulated AFOs was higher, whereas in SG3, with a lower QJS in both stances, the proportion of patients using non-articulated AFOs was higher. In SG2, with a lower QJS in the early stance and higher QJS in the middle stance, the proportion of patients using AFOs was lower. These findings indicate that classification of gait patterns based on QJS in patients after stroke may be helpful in selecting AFO. However, large sample sizes are required to confirm these results.

## 1. Introduction

In 2019, the global prevalence of stroke was 101.5 million people [1]. At the end of rehabilitation, approximately half of stroke patients achieve the ability to walk independently [2]. However, one-third of patients living in the community cannot safely walk outdoors after a stroke, limiting their ability to participate in community activities [3,4]. Strong predictors of community ambulation in patients after stroke are walking speed and walking endurance, which are related to forward propulsion [5,6,7]. Although an improvement in forward propulsion is necessary to increase community ambulation, ankle kinetics, which is an important factor for determining propulsion during gait, is often disturbed in patients after strokes [8,9].

An ankle foot orthosis (AFO) is a support device that can control the position and motion of the ankle by providing support for weak limbs or can position a limb into a more normal position. AFOs have been used in patients who have difficulty walking after stroke to improve gait patterns, gait speed, and energy expenditure [10,11,12]. An important characteristic of AFOs is stiffness, which involves a resistance to rotation at the ankle joint in the sagittal plane, as represented by the slope of the ankle moment versus the ankle angle curve of the AFO [13]. AFO stiffness in the dorsiflexion direction causes the storage of energy and return of energy in the late stance during gait, while AFO stiffness in the plantarflexion direction causes a limitation of the ankle plantarflexion angle [13,14]. A systematic review of AFO stiffness showed that increased AFO stiffness affected ankle kinematics (i.e., reduced peak ankle plantarflexion and increased dorsiflexion at initial contact) and knee kinematics (i.e., increased knee flexion at initial contact, reduced peak extension, and increased peak flexion in the stance phase) [13]. However, a systematic review of AFO use in patients after stroke did not show significant improvement in ankle kinetics after the use of AFOs [15,16]. Additionally, there is less evidence of AFO stiffness in ankle kinetics during gait in patients after stroke [13].

Ankle joint stiffness during gait, which is called quasi-joint stiffness (QJS), in patients after stroke is an important index for decision making regarding AFO stiffness. Our previous study revealed a significant association between QJS and ankle kinetics (i.e., peak ankle joint power at push off) on the paretic side in patients with hemiparesis [9]. However, QJS in the middle stance (MS) on the paretic side was lower than that on the non-paretic side and in controls [17,18]. Therefore, an increase of QJS in the MS pre-push-off with the paretic side in patients after stroke is important for decreased ankle joint power, to enhance force transmission during muscular contraction to the bone where the muscle inserts. In fact, increased AFO stiffness in the final range of motion of dorsiflexion, which is pre-push-off, when using a device with a cam-spring mechanism, assisted the generation of ankle joint power in the late stance in patients after stroke [19]. On the other hand, QJS in the early stance (ES) on the paretic side in patients after stroke was higher compared to that in healthy controls [18]. It may be also important to decrease QJS in the ES with AFO stiffness in the plantarflexion direction, because the ankle rocker plays a role in the forward rotation of the tibia, and the knee is not excessively extended in the ES in the gait of patients after stroke [20,21,22]. These facts indicate that clinicians should evaluate QJS in the ES and middle stance.

Many classifications based on gait pattern (i.e., gait kinematics) in patients after stroke have been proposed to guide clinicians when selecting the appropriate line of treatment. de Quervain et al. (1996) identified four patterns based on visual inspection of the knee joint angle on the paretic side in gait and gait speed in patients after stroke [23]. Kinsella et al. (2008) reported that the gait patterns of patients after stroke could be divided into three distinct patterns [24]. There were differences in ankle joint angles in the stance phase during gait among these groups [24]. Mulroy et al. (2003) demonstrated that the main factors classifying gait pattern were walking speed, peak knee extension in the MS, and peak dorsiflexion during swing [25]. Little suggested a classification based on pelvic excursion deviation in gait in patients after stroke [26]. Recently, Wang et al. (2021) revealed a classification based on abnormal gait kinematics (i.e., drop foot, circumduction, hip hiking, and back knee) in patients after stroke based on deep neural networks [27]. Thus, many studies have examined the classification of gait kinematics in patients after stroke. However, no study has developed a classification system based on gait kinetics like QJS in the ES and MS, which can provide more information for the selection of AFO stiffness. Information on patients with a high QJS in the ES and low QJS in the MS stance can help clinicians select AFO stiffness in the plantarflexion and dorsiflexion directions to reduce high QJS in the ES and increase low QJS in the MS stance. Additionally, it remains unclear whether there is an association between the classification based on QJS and a daily-used AFO, which has stiffness in both the plantarflexion and dorsiflexion directions (non-articulated AFO) or only in the plantarflexion direction (articulated AFO). Categorization based on QJS may help clinicians to appropriately select AFOs, which might result in the improvement of ankle kinetics and kinematics for patients after stroke.

The objectives of this study were to clarify (i) if the gait patterns of patients after stroke could be categorized based on QJS and (ii) differences in spatial and temporal parameters, joint kinematics and kinetics, and daily-use of AFO among these groups. In our two previous studies, the average of the coefficients of variation in the QJS in the ES and MS on the paretic side were higher than that of the non-paretic side [17,18]. Therefore, we had an a priori expectation of patients with various degrees of QJS in the ES and MS on the paretic side. Additionally, we hypothesized that in the subgroup with higher QJS in the ES, ankle plantarflexion and knee extension in the ES and the proportion of use of articulated or non-articulated AFOs, both of which have stiffness in the plantarflexion direction, would be higher than the other subgroups. Additionally, in the subgroup with lower QJS in the MS, ankle plantar flexion moment would be lower, and the proportion of use of non-articulated AFOs, which have stiffness in the dorsiflexion direction, would be higher than the other subgroups without this support.

## 2. Materials and Methods

### 2.1. Subjects

We recruited 72 patients with hemiplegia due to stroke. Inclusion criteria were as follows: (1) stroke due to ischemic or hemorrhagic supratentorial lesion, (2) stroke occurring at least one month before this study, (3) ability to walk a flat path of 7 m without the use of a cane and orthosis, and (4) ability to understand instructions by physical therapists. Exclusion criteria were as follows: (1) abnormal circulatory and respiratory status, (2) history of orthopedic problems that interfered with gait, (3) brainstem or cerebellar lesions, (4) diabetic neuropathy, (5) abnormal mental status, (6) higher brain dysfunction, which would cause skewed measurements, and (7) pain in the lower or upper limb when walking, which would cause skewed measurements. The present study was approved by the local ethics committee of Tohoku University of Medicine, Japan.

### 2.2. Clinical Characteristics

Information about clinical and demographic characteristics and daily-wear AFOs was obtained through interviews with the patients and from their medical records. The use of daily-wear AFOs for at least one week was divided into two types of AFO: articulated and non-articulated AFOs. All subjects who wore AFOs used passive devices, with reference to the previous study [15]. The number of subjects using articulated or non-articulated AFOs daily or not using AFOs was 24, 23, and 25, respectively. A physical therapist (Y.S.) evaluated the Brunnstrom Stages of Motor Recovery of the lower extremity and neurological impairment using the Stroke Impairment Assessment Set (SIAS) [28].

### 2.3. Motion Analysis

**Figure 1:**
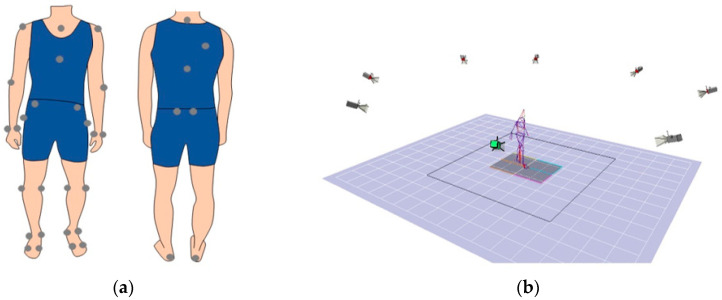
Reflective markers (*n* = 33) were attached to 12 segments and a three-dimensional motion analysis system. (**a**) The markers attached to 12 segments. (**b**) A 7 m gait was measured using an 8 camera motion analysis system and 4 force plates.

Three-dimensional coordinates of 33 reflective markers attached to 12 segments (Table 1) were measured using a three-dimensional motion analysis system (Motion Analysis Corporation, Santa, Rosa, CA, USA) equipped with eight cameras operating at 120 Hz. A 12-segment model based on anthropometric data was implemented as described by Dumas [29]. Ground reaction force (GRF) data were acquired at a 1200 Hz sampling rate using four 90 cm × 60 cm force plates (Anima Corporation, Chofu, Tokyo, Japan). The participants were asked to walk at a self-selected speed without assistance devices. All subjects walked 4–10 times on a 7 m long walkway until we successfully measured five steps on a unilateral side (i.e., a successful trial during which the foot strikes only one force plate and not two at the same time and the subjects do not stop, trip, or slip while walking), (Figure 1).

The three-dimensional coordinates and GRF data were smoothed using a bidirectional fourth-order Butterworth low-pass filter with a cutoff frequency of 6 Hz and 80 Hz, respectively. We decided the cutoff frequencies of three-dimensional coordinates based on a previous study [30]. The cutoff frequency of GRF was also determined with reference to a previous study [31]. For each joint in the lower extremities, kinematic data were calculated using a joint coordinate system [32]. In addition, the kinetics of the lower extremity joints were estimated using inverse dynamics [32]. All kinematic and kinetic data were time-normalized to 100% of the one-gait cycle. The representative parameters for the gait in the patient group were extracted from the kinematic and kinetic data following the method of a previous study [33]. The first and second maximum knee extension moment are the maximum knee extension moment in the first (i.e., in ES or MS) and second half of the stance phase (i.e., in MS or late stance). The representative parameters related to compensatory strategies in the paretic swing phase were calculated as the segment angle in the global coordination system [34]. The kinetic data were normalized to the patient’s body weight. The peak propulsion was identified as the maximum value of the GRF curve in the forward direction during gait.

We calculated QJS on the paretic side from the slope of the linear regression of the ankle joint moment versus the ankle angle during the second rocker interval, which was subdivided into ES and MS following the approach of previous studies [17,18]. We defined ES and MS in the second rocker interval as intervals from the peak plantarflexion angle in the stance of a leading (paretic) leg to the toe off of a trailing (non-paretic) leg and from the toe off of a trailing (non-paretic) leg to the maximum dorsiflexion angle in a stance of a leading (paretic) leg, respectively.

All data analyses were processed using a custom MATLAB program (MathWorks, Inc., Natick, MA, USA).

### 2.4. Statistics

A hierarchical cluster analysis was used to identify subgroups (SGs) with homogeneous QJS in ES and MS. In preparation for the cluster analysis, two clustering variables (i.e., only QJS in ES and MS but not in kinematic and kinetic parameters) were standardized using *Z*-scores; then, clustering algorithms utilizing Ward’s linkage method and squared Euclidean distance measures were applied. SGs were formed in an agglomerative manner, starting with each observation as their own SG and at every step pairing the two closest SGs together until only one group remained. The final number of SGs was chosen based on a stopping rule (a large percentage decrease in the coefficient followed by a plateau) [24]. The number of SGs was confirmed by visual inspection of the dendrogram [35].

Differences in age, height, weight, time post stroke, and gait spatiotemporal and kinematic and kinetic parameters were compared among SGs using one-way analysis of variance followed by Bonferroni’s multiple comparison procedure. Differences in sex, paretic side, and diagnosis among these groups were tested using Pearson’s chi-squared test. The Kruskal–Wallis non-parametric test, with the post hoc Wilcoxon rank sum test with Bonferroni correction, was used to compare the SAIS among SGs. Differences in the use of AFO among these groups were tested using two-sided Fisher’s exact test followed by one-sided Fisher’s exact test with Bonferroni corrections and mid-*p* adjustment. We used mid-*p* adjustment only when performing one-sided Fisher’s exact test for multiple comparison, because Fisher’s exact test is somewhat conservative [36,37]. A significance limit of α = 0.05 was selected. The eta-squared (η^2^) was calculated to estimate the effect size. Statistical analyses were performed using a statistical software package (SPSS Ver.24, IBM-SPSS Inc., Chicago, IL, USA) and JMP Pro 16 (SAS Institute Inc., Cary, NC, USA). JMP Pro 16 was used only when Fisher’s exact test was performed. In addition, one-sided Fisher’s exact test with mid-*p* adjustment and Bonferroni corrections was performed using a custom MATLAB program (MathWorks Inc., Natick, MA, USA) [38,39].

## 3. Results

**Figure 2:**
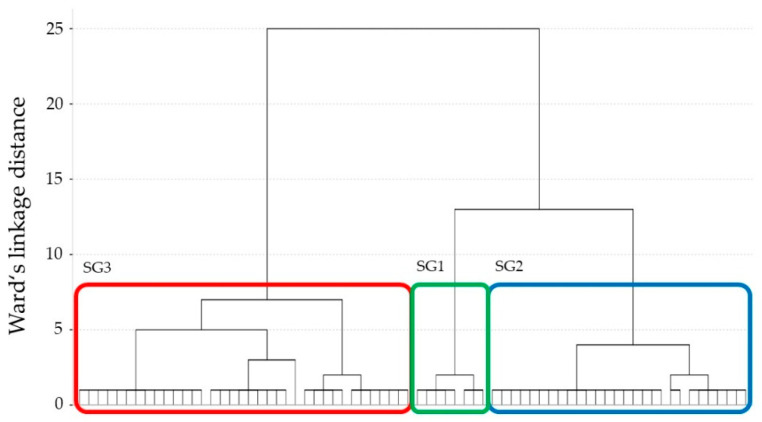
Dendrogram of the hierarchical cluster analysis. Linkage distance on the *y*-axis and individual patients on the *x*-axis. The three identified SGs (subgroups 1–3) are identified by a colored square.

The dendrogram for the clustering results is outlined in Figure 2. Between SGs two and three, there was a large decrease in the agglomeration coefficients (48.0%), followed by that between SGs three and four (33.0%). Additionally, the result was confirmed by visual inspection of the dendrogram, as shown in Figure 2. Therefore, the number of SGs was set to three.

### 3.1. Differences in Gait Parameters among the SGs

#### 3.1.1. Quasi-Joint Stiffness

**Figure 3:**
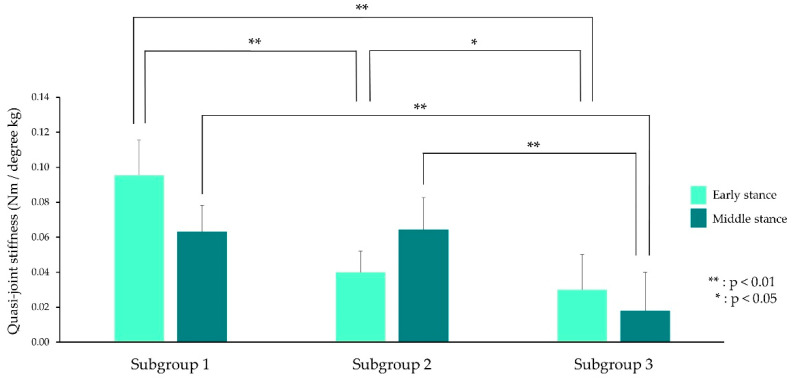
Quasi-joint stiffness in the early and middle stances in the three subgroups. Quasi-joint stiffness on the *y*-axis and individual subgroups on the *x*-axis. The light and dark green bars represents quasi-joint stiffness in the early and middle stances, respectively.

Figure 3 shows QJS in the subgroup 1 (SG1). There were significant differences in QJS in early (F_2,65_ = 50.542, *p* < 0.001, η^2^ = 0.59) and middle stances (F_2,65_ = 47.985, *p* < 0.001, η^2^ = 0.58) among the SGs.

QJS in the ES in SG1 was highest, although that in subgroup 2 (SG2) was the lowest among the SGs. On the other hand, the QJS in middle stance in subgroup 3 (SG3) was lower than that in SG1 (*p* < 0.001) and SG2 (*p* < 0.001).

#### 3.1.2. Spatiotemporal Parameters

Table 2 shows the spatiotemporal parameters in the three SGs. There were differences in gait speed (F_2,65_ = 25.999, *p* < 0.001, η^2^ = 0.43), cadence (F_2,65_ = 16.969, *p* < 0.001, η^2^ = 0.33), stance time (F_2,65_ = 9.084, *p* < 0.001, η^2^ = 0.21), swing time (F_2,65_ = 6.695, *p* = 0.002, η^2^ = 0.16), single stance time (F_2,65_ = 5.057, *p* = 0.009, η^2^ = 0.13), initial (F_2,65_ = 11.263, *p* < 0.001, η^2^ = 0.25) and late double stance time (F_2,65_ = 8.613, *p* < 0.001, η^2^ = 0.20), paretic step length (F_2,65_ = 18.525, *p* < 0.001, η^2^ = 0.35), and non-paretic step length (F_2,65_ = 25.974, *p* < 0.001, η^2^ = 0.43).

SG3 had the slowest gait speed (SG3 vs. SG2: *p* < 0.001, SG3 vs. SG1: *p* < 0.001) and cadence (SG3 vs. SG2: *p* < 0.001, SG3 vs. SG1: *p* < 0.001), longest stance time (SG3 vs. SG2: *p* = 0.001, SG3 vs. SG1: *p* = 0.019) and initial double stance time (SG3 vs. SG2: *p* < 0.001, SG3 vs. SG1: *p* = 0.010), and shortest paretic (SG3 vs. SG2: *p* < 0.001, SG3 vs. SG1: *p* = 0.001) and non-paretic step lengths (SG3 vs. SG2: *p* < 0.001, SG3 vs. SG1: *p* = 0.001) among the three SGs. Additionally, there were no significant differences between SG1 and SG2 for any of these parameters.

#### 3.1.3. Kinematic Parameters

Table 3 shows the representative kinematic parameters in three SGs. There were significant differences in knee flexion at heel contact (F_2,65_ = 3.417, *p* = 0.038, η^2^ = 0.09) and toe off (F_2,65_ = 10.849, *p* < 0.001, η^2^ = 0.24), maximum knee flexion in swing (F_2,65_ = 14.455, *p* < 0.001, η^2^ = 0.30), knee (F_2,65_ = 11.205, *p* < 0.001, η^2^ = 0.25) and hip angle excursion (F_2,65_ = 20.642, *p* < 0.001, η^2^ = 0.37), hip flexion at heel contact (F_2,65_ = 3.312, *p* = 0.042, η^2^ = 0.09), maximum hip extension in stance (F_2,65_ = 8.726, *p* < 0.001, η^2^ = 0.20), and hip flexion at toe off (F_2,65_ = 3.731, *p* = 0.029, η^2^ = 0.10), but not in the kinematic parameters related to the ankle joint. Moreover, there tended to be a difference in maximum knee flexion in the ES (F_2,65_ = 3.085, *p* = 0.052, η^2^ = 0.08) and knee extension in stance (F_2,65_ = 2.856, *p* = 0.064, η^2^ = 0.08).

A lower maximum knee flexion in the swing phase (SG3 vs. SG2: *p* < 0.001, SG3 vs. SG1: *p* = 0.044) and knee (SG3 vs. SG2: *p* < 0.001, SG3 vs. SG1: *p* = 0.019) and hip angle excursion (SG3 vs. SG2: *p* < 0.001, SG3 vs. SG1: *p* = 0.001) were found in SG3 compared with the other SGs. In SG2, the maximum hip extension in stance (*p =* 0.001), hip flexion at push off (*p =* 0.037), maximum knee flexion in the ES (*p =* 0.050), and knee flexion at push off (*p <* 0.001) were significantly larger than that of SG3. However, there was no significant difference in any kinematic parameters between SG1 and SG2.

#### 3.1.4. Kinetic Parameters

Table 4 shows the representative kinetic parameters for the three SGs. There were significant differences in maximum hip extension moment in ES (F_2,65_ = 7.235, *p* = 0.001, η^2^ = 0.17), maximum hip flexion moment in stance (F_2,65_ = 12.048, *p* < 0.001, η^2^ = 0.26), first maximum knee extension moment (F_2,65_ = 3.607, *p* = 0.032, η^2^ = 0.10), maximum ankle dorsiflexion moment in ES (F_2,65_ = 6.024, *p* = 0.004, η^2^ = 0.15), and maximum ankle plantarflexion moment in stance (F_2,65_ = 47.293, *p* < 0.001, η^2^ = 0.58).

Among the three SGs, the lowest maximum ankle plantarflexion (SG3 vs. SG2: *p* < 0.001, SG3 vs. SG1: *p* < 0.001) and dorsiflexion moment (SG3 vs. SG2: *p* = 0.048, SG3 vs. SG1: *p* = 0.010) were found in SG3. In SG2, maximum hip flexion moment (*p* < 0.001) and the first maximum knee extension moment (*p* = 0.029) were larger than those in SG3. In SG1, the maximum hip extension moment in ES was larger than those in SG3 (*p* = 0.002).

### 3.2. Differences in Demographic Data and Clinical Features among the Subgroups

#### 3.2.1. Demographic Data

No differences (all *p* > 0.156) in age, height, weight, time post stroke, sex, diagnosis, and paretic side among the SGs were found, as shown in Table 5.

#### 3.2.2. Clinical Features

In SG3, items for motor function in lower limb in the SIAS were lower than those in SG2 (ankle joint: *p* = 0.002, knee joint: *p* = 0.009, and hip joint: *p* = 0.004), as shown in Table 5. However, there was no significant difference in deep tendon reflex and range of motion in ankle dorsiflexion in SIAS among the SGs.

#### 3.2.3. Use of AFOs

Table 6 shows the distribution of use of AFOs in the three groups. We found statistically significant differences in the non-use of AFOs, use of articulated AFOs, and use of non-articulated AFOs among the three SGs. The proportion of patients without AFOs in SG2 was significantly higher than that in SG3 (*p* = 0.008). The proportion of patients with articulated AFOs in SG1 was significantly higher than that in SG2 (*p* = 0.040). The proportion of patients with non-articulated AFOs in SG3 was significantly higher than that in SG1 (*p* = 0.036).

Participants who used the articulated AFOs daily wore an AFO with resistance in the plantarflexion direction due to an oil damper and without stiffness in the dorsiflexion direction (Gait Solution Design; Kawamura Gishi, Osaka, Japan) or a posterior leaf spring plastic AFO with a Tamarack flexure joint (Becker Orthopedic, Troy, MI, USA). The participants who used non-articulated AFOs daily wore an ORTOP AFO (Pacific Supply Co., Ltd., Osaka, Japan) or posterior leaf spring plastic AFO.

## 4. Discussion

We assessed the gait pattern based on ankle joint stiffness, also called QJS, in the gait in patients with hemiparesis using a hierarchical cluster analysis. The group was divided into three SGs, with different gait patterns based on knee kinematics, especially maximum knee extension in MS, as described in previous studies [23,24,25,40]. However, interestingly, the three groups had different proportions of AFO use, which supports our hypothesis. This is the first study that demonstrates that the type of QJS in the gait of patients after stroke can determine whether they use articulated AFOs, non-articulated AFOs, or no AFO.

The highest QJS in the ES on the paretic side was found in SG1. However, the lower limb reflex, including Achilles’ tendon reflex and passive dorsiflexion range of motion at the ankle joint, did not differ from that of the other SGs. As indicated in previous studies, QJS in the ES may contribute to other factors, such as viscous damping at the ankle joint, but not the dorsiflexion range of motion and increased muscle activity at the ankle joint in the ES due to spasticity [18,41]. Contrary to our hypothesis, the group with a high QJS in the ES (SG1) did not exhibit hyperextension of the knee in the ES. This is because dorsiflexion movement in the ES was not low, which may be a stronger factor preventing hyper knee extension than QJS in the ES [42]. Additionally, higher hip extension moment in the ES on the paretic side was found in SG1. The impaired ankle rocker in the ES due to high QJS may not play a role in moving the center of mass (COM) in the forward direction, which has been shown as an inverted pendulum model [43]. Because gait speed in SG1 did not decrease compared to that of the other SGs, the increased hip extension moment on the paretic side may have compensated for decreased forward COM movement due to high QJS in the ES. QJS in the MS in SG1 was also higher than in SG3, which resulted in higher ankle plantarflexion moment in stance, as was predicted by our hypothesis. SG1 does not belong to any of the groups that were presented in previous studies [23,24,25]. The proportion of patients with articulated AFOs in SG1 was significantly higher than in SG2; while that with non-articulated AFOs in SG1 was significantly lower in SG3. Articulated AFOs, which were used by patients in SG1, were associated with AFO stiffness in the plantarflexion direction but not AFO stiffness in dorsiflexion [44]. Patients in SG1 may not need an increase in QJS in the MS due to AFO stiffness in the dorsiflexion direction, which the non-articulated AFO has, although they need a decrease in QJS in the ES due to AFO stiffness in the plantarflexion direction, which the articulated AFO has. However, care should be taken in interpreting the result regarding the proportion of patients with articulated AFOs in SG1 due to the small number of subjects in that group.

SGs 1 and 2 showed a higher QJS in the MS compared to SG3, and SG2 showed the second highest QJS in the ES. Additionally, the patients had milder paresis in the lower limb on the paretic side than in SG3. Therefore, in patients with high motor function in the lower limbs, the characteristics of QJS were similar to those in the healthy controls, such as low QJS in the ES and high QJS in the MS, as shown in a previous study [18]. Due to the high QJS in the MS, the high ankle plantarflexion moment in the stance phase may cause a higher gait speed in SG2, which was consistent with previous findings [9,18], and a high proportion of the non-use of AFOs. However, knee flexion throughout the gait cycle in SG2 increased. The maximum knee extension in the stance phase (−6.3 ± 6.1) in SG2 was similar to that reported by Kinsella for SG2 (−2.3 ± 4.0) [24]. High knee flexion in the early stance during weight transfer was similar to the buckling knee pattern with intermediate gait speed, as reported by de Quervain [23]. Kobayashi reported that AFO stiffness in the dorsiflexion direction increased knee extension in the stance phase in patients after stroke [45]. These facts indicate that the non-use of AFOs may lead to excessive knee flexion in ES in patients after stroke. In addition, the second highest QJS in ES, in which the direction of resistance is consistent with AFO stiffness in the dorsiflexion direction, may prevent excessive knee flexion in the ES in a buckling knee pattern.

The lowest QJS in the ES and MS on the paretic side were observed in SG3. Additionally, evaluations of motor function in the lower limb showed severe paresis in the lower limb in SG3. Severe paresis may decrease the activity of the ankle plantar flexor muscle in the MS, thus resulting in a decrease of QJS in the MS, as shown in a previous study [18]. Because there was no difference in reflexes in the lower limb and passive ankle dorsiflexion range of motion among the SGs, which has an effect on QJS, severe paresis may be a main factor decreasing QJS in the ES. The results of lower limb kinematics in SG3 showed decreased knee flexion in the ES at push off, swing and hip extension in stance, and increased hip flexion and compensation movements in swing. Decreased QJS in the ES may cause decreased knee flexion in the ES due to poor eccentric control of the tibia. On the other hand, the low QJS in the MS may decrease the ankle plantarflexion moment, thus resulting in insufficient knee flexion at toe off and swing and excessive hip flexion at toe off and compensation movements in swing, as shown in previous studies [9,18,46,47,48]. The proportion of use of non-articulated AFOs in SG3 was higher, whereas a previous study showed that AFO with severe stiffness in the plantarflexion direction reduced ankle joint power generation at push off in the gait of patients after stroke [49]. It seemed to be unreasonable that patients in SG3, who had a decrease in ankle kinetics at push off, used non-articulated AFOs to reduce ankle joint power generation at push off. Tsuchiyama et al. (2021) reported that in groups with severe ankle impairment, the use of AFOs improved stability in the gait of patients after stroke [50]. Therefore, our results indicate that SG3, with the lowest QJS in ES and MS, may need more stability by using non-articulated AFOs with severe stiffness in the dorsiflexion and plantarflexion directions, due to poor control of the tibia rather than propulsion by push off.

There were several limitations of the present study. First, only patients who could walk without any assistance after stroke were recruited. Patients with a slow gait speed in the previous studies used a four-point or straight cane when walking [23,25]. Additionally, in comparison to those in the previous studies (Kinsella et al. (2008), 0.13 m/s and 0.23 m/s; De Quervain et al. (1996), 0.16 m/s), the gait speed (0.31 m/s) in the SGs that was categorized as slow gait speed in the present study was faster [23,24]. The differences in gait function between the present study and previous studies may have an effect on the difference in categorization based on knee kinematics at slow gait speed. Second, the present study did not measure gait with daily-used AFOs. Therefore, it is unclear whether daily-used AFOs are the most appropriate. Third, SG3 did not have any features of maximum knee extension in the stance phase (−1.8 ± 9.4°) with a slow gait speed. Previous studies showed that there were two types of maximum knee extension (i.e., excessive knee flexion and extension) in groups with slow gait speed [24,25]. There was no difference in plantar flexor activity or ankle plantarflexion moment in the stance phase between the SGs with two knee types in previous studies [24,25]. The categorization in this study is based on QJS, which is attributed to ankle plantarflexion activity and moment in the stance phase during gait. Therefore, SG3, with a slow gait speed, was not divided into two knee types as in previous studies [24,25]. Fourth, the present study was cross-sectional. Future research should prospectively follow patients after stroke to clarify whether similar SGs exist prior to determining daily-used AFOs. Fifth, the sample size of SG1 was small. A future study to measure and analyze QJS in a larger sample size is warranted.

Future research should collect both kinetic/QJS and kinematic data for gait classification. However, appropriate data (i.e., kinematic parameters, other parameters) for use in gait classification may depend on the purpose for which the gait classification is used. A variety of data on gait in stroke patients is needed to determine a gait classification index for appropriate treatment and orthotic selection. In our present results, the SG1 sample size was small. The use of 3D motion analysis is limited in terms of resources to measure large amounts of data, which may require the use of wearable sensors or depth sensors that are currently being developed. In addition, future research on gait classification, including patients in the subacute phase, should provide valuable information to clarify the recovery process.

## 5. Conclusions

In summary, we classified the gait pattern based on ankle joint stiffness, called QJS, in the gait of patients after stroke and clarified the differences in the use of AFOs among the groups as follows: the proportion of use of articulated AFOs, that of non-articulated AFOs, and the non-use of AFOs were higher in SG1, with a high QJS in the ES and MS, followed by SG3, with a low QJS in the ES and MS, and SG2, with a low QJS in the ES and high QJS in the MS. However, large sample sizes are required to confirm these results.

## Figures and Tables

**Table 1:** Placement of markers on the body that defined the 12 segments of the motion capture kinematic model.

Segment	Placement of Markers
Trunk	Spinous process of the 7th cervical vertebrae, spinous process of the 10th thoracic vertebrae, jugular notch where the clavicles meet the sternum, xiphoid process of the sternum, and the position in the middle of the right scapula
Upper arm	Both acromions and both lateral epicondyles of elbow
Forearm	Both lateral epicondyles of the elbow and both styloid processes of the ulna and radius
Pelvis	Both anterior superior iliac spines and both posterior superior iliac spines
Thigh	Both greater trochanters and both lateral and medial epicondyles of knee
Shank	Both lateral epicondyles of knee and both lateral and medial malleolus
Foot	Both the first and fifth metatarsal heads, both lateral and medial malleolus, and both calcaneus

**Table 2:** Gait spatiotemporal parameters measured using a three-dimensional motion analysis system in the three subgroups.

		Group			Statistics					
		SG1	SG2	SG3	ANOVA			*p*-Values of the Post Hoc Test		
					*F*-Value	*p*-Value	η^2^	SG1 vs. SG2	SG1 vs. SG3	SG2 vs. SG3
Stance time (s)	Mean	0.78	0.84	1.31	9.084	**<0.001**	0.208	1.000	**0.019**	**0.001**
	SD	0.13	0.14	0.66						
Swing time (s)	Mean	0.50	0.51	0.60	6.695	**0.002**	0.163	1.000	0.069	**0.004**
	SD	0.06	0.09	0.12						
Single stance time (s)	Mean	0.38	0.43	0.34	5.057	**0.009**	0.128	0.763	1.000	**0.007**
	SD	0.08	0.05	0.14						
Initial double stance time (s)	SD	0.17	0.20	0.53	11.263	**<0.001**	0.246	1.000	**0.010**	**<0.001**
	SD	0.07	0.07	0.42						
Late double stance time (s)	Mean	0.24	0.22	0.44	8.613	**<0.001**	0.200	1.000	0.065	**0.001**
	SD	0.16	0.09	0.29						
Walking speed (m/s)	Mean	0.70	0.67	0.31	25.99	**<0.001**	0.430	1.000	**<0.001**	**<0.001**
	SD	0.31	0.23	0.18						
Step length (m)										
Paretic side	Mean	0.44	0.43	0.27	18.525	**<0.001**	0.349	1.000	**0.001**	**<0.001**
	SD	0.12	0.11	0.13						
Non-paretic side	Mean	0.39	0.42	0.22	25.974	**<0.001**	0.430	1.000	**0.001**	**<0.001**
	SD	0.17	0.10	0.11						
Stride length (m)	Mean	0.86	0.87	0.51	25.410	**<0.001**	0.424	1.000	**<0.001**	**<0.001**
	SD	0.28	0.21	0.20						
Cadence (steps/min)	Mean	95.55	91.05	69.23	16.969	**<0.001**	0.330	1.000	**<0.001**	**<0.001**
	SD	14.74	13.93	18.93						
Step width (m)	Mean	0.15	0.14	0.18	6.944	**0.002**	0.168	1.000	0.120	**0.002**
	SD	0.04	0.03	0.05						

SG: subgroup; ANOVA: analysis of variance; *p*-values marked with bold indicate statistically significant differences at the *p* < 0.05 level.

**Table 3:** Representative kinematic parameters in paretic lower limb during walk in the three subgroups.

		Group			Statistics					
		SG1	SG2	SG3	ANOVA			*p*-Values of the Post Hoc Test		
					*F*-Value	*p*-Value	η^2^	SG1 vs. SG2	SG1 vs. SG3	SG2 vs. SG3
Maximum ankle plantarflexion in early stance (°)	Mean	1.49	3.32	6.01	2.304	0.107	0.063	1.000	0.236	0.312
	SD	5.93	5.18	7.42						
Maximum ankle dorsiflexion in stance (°)	Mean	−12.01	−15.14	−12.45	1.461	0.239	0.041	0.752	1.000	0.353
	SD	7.73	4.63	7.82						
Ankle plantarflexion at toe off (°)	Mean	1.82	1.00	−1.12	0.756	0.473	0.021	1.000	1.000	0.912
	SD	9.54	7.56	8.17						
Maximum ankle dorsiflexion in swing (°)	Mean	−5.09	−5.17	−2.77	1.027	0.364	0.029	1.000	1.000	0.544
	SD	7.20	5.85	7.84						
Ankle angle excursion (°)	Mean	17.51	21.99	23.84	2.255	0.113	0.061	0.460	0.121	1.000
	SD	6.72	6.74	8.62						
Knee flexion at heel contact (°)	Mean	−10.97	−12.26	−7.50	3.417	**0.038**	0.090	1.000	0.696	**0.037**
	SD	5.08	4.81	9.17						
Maximum knee flexion in early stance (°)	Mean	−15.63	−17.61	−11.83	3.085	0.052	0.082	1.000	0.904	**0.050**
	SD	5.26	6.08	11.75						
Maximum knee extension in stance (°)	Mean	−1.21	−6.26	−1.82	2.856	0.064	0.076	0.346	1.000	0.087
	SD	5.47	6.08	9.40						
Knee flexion at toe off (°)	Mean	−27.59	−31.26	−19.17	10.849	**<0.001**	0.236	1.000	0.129	**<0.001**
	SD	10.23	7.26	12.41						
Maximum knee flexion in swing (°)	Mean	−39.07	−43.64	−26.36	14.455	**<0.001**	0.295	1.000	**0.044**	**<0.001**
	SD	13.55	10.74	14.40						
Knee angle excursion (°)	Mean	38.21	38.48	25.57	11.205	**<0.001**	0.245	1.000	**0.019**	**<0.001**
	SD	13.20	11.11	11.47						
Hip flexion at heel contact (°)	Mean	−28.88	−28.64	−23.84	3.312	**0.042**	0.088	1.000	0.337	0.060
	SD	4.21	7.91	8.65						
Maximum hip extension in stance (°)	Mean	−2.37	−1.85	−9.91	8.726	**<0.001**	0.202	1.000	0.059	**0.001**
	SD	8.19	7.62	8.38						
Hip flexion at toe off (°)	Mean	−9.79	−9.67	−15.07	3.731	**0.029**	0.098	1.000	0.330	**0.037**
	SD	7.87	8.15	8.59						
Maximum hip flexion during swing (°) Hip angle excursion (°)	Mean	−33.35	−32.72	−28.89	2.238	0.114	0.061	1.000	0.475	0.184
	SD	4.95	7.37	8.91						
	Mean	31.04	31.13	19.74	20.642	**<0.001**	0.374	1.000	**0.001**	**<0.001**
	SD	7.99	6.45	8.14						
Pelvis hiking (°)	Mean	5.06	3.10	5.30	3.280	**0.044**	0.087	0.499	1.000	**0.044**
	SD	2.28	3.40	3.75						
Leg circumduction (°)	Mean	11.54	9.58	13.01	4.985	**0.010**	0.126	0.786	1.000	**0.007**
	SD	5.19	3.83	4.49						

SG: subgroup; ANOVA: analysis of variance; Negative value represents flexion or dorsiflexion; *p*-values marked with bold indicate statistically significant differences at the *p* < 0.05 level.

**Table 4:** Representative kinetic parameters in paretic lower limb during walk in the three subgroups.

		Group			Statistics					
		SG1	SG2	SG3	ANOVA			*p*-Values of the Post Hoc Test		
					*F*-Value	*p*-Value	η^2^	SG1 vs. SG2	SG1 vs. SG3	SG2 vs. SG3
Maximum hip extension moment in early stance (Nm/kg)	Mean	0.84	0.61	0.47	7.235	**0.001**	0.173	0.085	**0.002**	0.123
	SD	0.12	0.30	0.25						
Maximum hip flexion moment in stance phase (Nm/kg)	Mean	−0.52	−0.60	−0.33	12.048	**<0.001**	0.259	1.000	0.098	**<0.001**
	SD	0.30	0.21	0.20						
First maximum knee extension moment in stance phase (Nm/kg)	Mean	0.27	0.33	0.18	3.607	**0.032**	0.095	1.000	0.798	**0.029**
	SD	0.23	0.18	0.23						
Maximum knee flexion moment in stance phase (Nm/kg)	Mean	−0.36	−0.05	−0.13	2.265	0.112	0.062	0.112	0.336	1.000
	SD	0.31	0.29	0.43						
Second maximum knee extension moment in stance phase (Nm/kg)	Mean	0.17	0.28	0.22	1.272	0.287	0.036	0.530	1.000	0.642
	SD	0.14	0.16	0.23						
Maximum ankle dorsiflexion moment in early stance (Nm/kg)	Mean	−0.06	−0.04	−0.01	6.024	**0.004**	0.149	0.487	**0.010**	**0.048**
	SD	0.07	0.04	0.04						
Maximum ankle plantarflexion moment in stance phase (Nm/kg)	Mean	1.01	0.97	0.53	47.293	**<0.001**	0.578	1.000	**<0.001**	**<0.001**
	SD	0.16	0.20	0.19						

SG: subgroup; ANOVA: analysis of variance; *p*-values marked with bold indicate statistically significant differences at the *p* < 0.05 level.

**Table 5:** Demographic and clinical features of patients in the three subgroups.

	Group		
	SG1 ^1^	SG2	SG3
Number ^2^	8	28	36
Gender (Male/Female) ^2^	6/2	22/6	25/11
Age (years) ^3^	52.6(9.5)	55.7(12.2)	58.5(11.4)
Height (cm) ^3^	162.1(5.6)	167.8(9.0)	164.9(7.6)
Weight (kg) ^3^	62.1(8.4)	65.8(10.6)	61.7(11.9)
Diagnosis (Hemorrhage/Infarction) ^2^	6/2	18/10	18/18
Paretic side (Right/Left) ^2^	3/5	17/11	21/15
Time since neurologic event (month) ^3^	11.25(18.35)	35.82(40.98)	36.31(49.49)
SIAS ^2,4^			
Motor function (0/1/2/3/4/5)			
Ankle joint	1/2/0/2/3/0	1/3/1/7/13/3	5/9/5/11/5/1
Knee joint	0/0/0/3/4/1	0/0/2/7/15/4	0/2/3/21/8/2
Hip joint	0/0/0/3/5/0	0/0/1/4/19/4	0/1/3/16/15/1
Deep tendon reflex (lower limb) (0/1/2/3)	0/5/2/1	1/10/8/9	1/13/18/4
Range of motion (ankle dorsiflexion) (0/1/2/3)	1/5/2/0	2/11/13/2	1/19/14/2

^1^ SG: subgroup; ^2^ Number of patients; ^3^ Mean (standard deviation); ^4^ SIAS: stroke impairment assessment set.

**Table 6:** The ratio and number of subjects who used AFOs daily in the three subgroups.

	SG1 (*n* = 8)		SG2 (*n* = 28)		SG3 (*n* = 36)		*p*-Value SG 1 vs. 2	SG 1 vs. 3	SG 2 vs. 3
	*n*	%	*n*	%	*n*	%			
Non-use of AFO	3	38	15	54	7	19	0.689	0.475	**0.008**
Use of articulate AFO	5	63	5	18	14	39	**0.040**	0.387	0.113
Use of non-articulate AFO	0	0	8	29	15	42	0.154	**0.036**	0.442

AFO: ankle foot orthosis; SG: subgroup; *p*-values marked with bold indicate statistically significant differences at the *p* < 0.05 level.

## Data Availability

Data are available on request due to privacy and ethical restrictions.

## References

[1] Virani S.S., Alonso A., Aparicio H.J., Benjamin E.J., Bittencourt M.S., Callaway C.W., Carson A.P., Chamberlain A.M., Cheng S., Delling F.N. (2021). Heart Disease and Stroke Statistics-2021 Update: A Report from the American Heart Association. Circulation.

[2] Jørgensen H.S., Nakayama H., Raaschou H.O., Olsen T.S. (1995). Recovery of walking function in stroke patients: The Copenhagen Stroke Study. Arch. Phys. Med. Rehabil..

[3] Lord S.E., McPherson K., McNaughton H.K., Rochester L., Weatherall M. (2004). Community ambulation after stroke: How important and obtainable is it and what measures appear predictive?. Arch. Phys. Med. Rehabil..

[4] Barclay R.E., Stevenson T.J., Poluha W., Ripat J., Nett C., Srikesavan C.S. (2015). Interventions for improving community ambulation in individuals with stroke. Cochrane Database Syst. Rev..

[5] Awad L.N., Binder-Macleod S.A., Pohlig R.T., Reisman D.S. (2015). Paretic Propulsion and Trailing Limb Angle Are Key Determinants of Long-Distance Walking Function After Stroke. Neurorehabil. Neural Repair.

[6] Fulk G.D., He Y., Boyne P., Dunning K. (2017). Predicting Home and Community Walking Activity Poststroke. Stroke.

[7] Roelker S.A., Bowden M.G., Kautz S.A., Neptune R.R. (2019). Paretic propulsion as a measure of walking performance and functional motor recovery post-stroke: A review. Gait Posture.

[8] Hsiao H., Knarr B.A., Higginson J.S., Binder-Macleod S.A. (2015). Mechanisms to increase propulsive force for individuals poststroke. J. Neuroeng. Rehabil..

[9] Sekiguchi Y., Muraki T., Kuramatsu Y., Furusawa Y., Izumi S. (2012). The contribution of quasi-joint stiffness of the ankle joint to gait in patients with hemiparesis. Clin. Biomech..

[10] Daryabor A., Yamamoto S., Orendurff M., Kobayashi T. (2020). Effect of types of ankle-foot orthoses on energy expenditure metrics during walking in individuals with stroke: A systematic review. Disabil. Rehabil..

[11] Wada Y., Otaka Y., Mukaino M., Tsujimoto Y., Shiroshita A., Kawate N., Taito S. (2021). The effect of ankle-foot orthosis on ankle kinematics in stroke individuals: A systematic review and meta-analysis. PMR J. Inj. Funct. Rehabil..

[12] Choo Y.J., Chang M.C. (2021). Effectiveness of an ankle-foot orthosis on walking in patients with stroke: A systematic review and meta-analysis. Sci. Rep..

[13] Totah D., Menon M., Jones-Hershinow C., Barton K., Gates D.H. (2019). The impact of ankle-foot orthosis stiffness on gait: A systematic literature review. Gait Posture.

[14] Bregman D.J., Harlaar J., Meskers C.G., de Groot V. (2012). Spring-like Ankle Foot Orthoses reduce the energy cost of walking by taking over ankle work. Gait Posture.

[15] Daryabor A., Arazpour M., Aminian G. (2018). Effect of different designs of ankle-foot orthoses on gait in patients with stroke: A systematic review. Gait Posture.

[16] Tyson S.F., Sadeghi-Demneh E., Nester C.J. (2013). A systematic review and meta-analysis of the effect of an ankle-foot orthosis on gait biomechanics after stroke. Clin. Rehabil..

[17] Sekiguchi Y., Muraki T., Owaki D., Honda K., Izumi S.I. (2018). Regulation of quasi-joint stiffness by combination of activation of ankle muscles in midstances during gait in patients with hemiparesis. Gait Posture.

[18] Sekiguchi Y., Muraki T., Tanaka N., Izumi S. (2015). Relationship between activation of ankle muscles and quasi-joint stiffness in early and middle stances during gait in patients with hemiparesis. Gait Posture.

[19] Sekiguchi Y., Owaki D., Honda K., Fukushi K., Hiroi N., Nozaki T., Izumi S.I. (2020). Ankle-foot orthosis with dorsiflexion resistance using spring-cam mechanism increases knee flexion in the swing phase during walking in stroke patients with hemiplegia. Gait Posture.

[20] Perry J., Schoneberger B. (1992). GAIT ANALYSIS: Normal and Pathological Function.

[21] Kobayashi T., Leung A.K., Akazawa Y., Hutchins S.W. (2013). The effect of varying the plantarflexion resistance of an ankle-foot orthosis on knee joint kinematics in patients with stroke. Gait Posture.

[22] Kobayashi T., Singer M.L., Orendurff M.S., Gao F., Daly W.K., Foreman K.B. (2015). The effect of changing plantarflexion resistive moment of an articulated ankle-foot orthosis on ankle and knee joint angles and moments while walking in patients post stroke. Clin. Biomech..

[23] De Quervain I.A., Simon S.R., Leurgans S., Pease W.S., McAllister D. (1996). Gait pattern in the early recovery period after stroke. J. Bone Joint Surg. Am..

[24] Kinsella S., Moran K. (2008). Gait pattern categorization of stroke participants with equinus deformity of the foot. Gait Posture.

[25] Mulroy S., Gronley J., Weiss W., Newsam C., Perry J. (2003). Use of cluster analysis for gait pattern classification of patients in the early and late recovery phases following stroke. Gait Posture.

[26] Little V.L., McGuirk T.E., Perry L.A., Patten C. (2018). Pelvic excursion during walking post-stroke: A novel classification system. Gait Posture.

[27] Wang F.C., Chen S.F., Lin C.H., Shih C.J., Lin A.C., Yuan W., Li Y.C., Kuo T.Y. (2021). Detection and Classification of Stroke Gaits by Deep Neural Networks Employing Inertial Measurement Units. Sensors.

[28] Tsuji T., Liu M., Sonoda S., Domen K., Chino N. (2000). The stroke impairment assessment set: Its internal consistency and predictive validity. Arch. Phys. Med. Rehabil..

[29] Dumas R., Cheze L., Verriest J.P. (2007). Adjustments to McConville et al. and Young et al. body segment inertial parameters. J. Biomech..

[30] Honda K., Sekiguchi Y., Muraki T., Izumi S.I. (2019). The differences in sagittal plane whole-body angular momentum during gait between patients with hemiparesis and healthy people. J. Biomech..

[31] Luc-Harkey B.A., Franz J.R., Blackburn J.T., Padua D.A., Hackney A.C., Pietrosimone B. (2018). Real-time biofeedback can increase and decrease vertical ground reaction force, knee flexion excursion, and knee extension moment during walking in individuals with anterior cruciate ligament reconstruction. J. Biomech..

[32] Robertson G.E., Caldwell G.E., Hamill J., Kamen G., Whittlesey S. (2013). Research Methods in Biomechanics.

[33] Benedetti M.G., Catani F., Leardini A., Pignotti E., Giannini S. (1998). Data management in gait analysis for clinical applications. Clin. Biomech..

[34] Kerrigan D.C., Frates E.P., Rogan S., Riley P.O. (2000). Hip hiking and circumduction: Quantitative definitions. Am. J. Phys. Med. Rehabil..

[35] Jauhiainen S., Pohl A.J., Äyrämö S., Kauppi J.P., Ferber R. (2020). A hierarchical cluster analysis to determine whether injured runners exhibit similar kinematic gait patterns. Scand. J. Med. Sci. Sports.

[36] Lydersen S., Pradhan V., Senchaudhuri P., Laake P. (2007). Choice of test for association in small sample unordered r × c tables. Stat. Med..

[37] Lydersen S., Fagerland M.W., Laake P. (2009). Recommended tests for association in 2 × 2 tables. Stat. Med..

[38] Thorvaldsen S., Flå T., Willassen N.P. (2010). DeltaProt: A software toolbox for comparative genomics. BMC Bioinform..

[39] Thorvaldsen S. (2010). Fisher’s Exact with Mid-P Method. https://jp.mathworks.com/matlabcentral/fileexchange/29819-fisher-s-exact-with-mid-p-method.

[40] Kaczmarczyk K., Wit A., Krawczyk M., Zaborski J. (2009). Gait classification in post-stroke patients using artificial neural networks. Gait Posture.

[41] Shorter A.L., Richardson J.K., Finucane S.B., Joshi V., Gordon K., Rouse E.J. (2021). Characterization and clinical implications of ankle impedance during walking in chronic stroke. Sci. Rep..

[42] Cooper A., Alghamdi G.A., Alghamdi M.A., Altowaijri A., Richardson S. (2012). The relationship of lower limb muscle strength and knee joint hyperextension during the stance phase of gait in hemiparetic stroke patients. Physiother. Res. Int..

[43] Kuo A., Donelan J., Ruina A. (2005). Energetic consequences of walking like an inverted pendulum: Step-to-step transitions. Exerc. Sport Sci. Rev..

[44] Ohata K., Yasui T., Tsuboyama T., Ichihashi N. (2011). Effects of an ankle-foot orthosis with oil damper on muscle activity in adults after stroke. Gait Posture.

[45] Kobayashi T., Orendurff M.S., Hunt G., Lincoln L.S., Gao F., LeCursi N., Foreman K.B. (2017). An articulated ankle-foot orthosis with adjustable plantarflexion resistance, dorsiflexion resistance and alignment: A pilot study on mechanical properties and effects on stroke hemiparetic gait. Med. Eng. Phys..

[46] Campanini I., Merlo A., Damiano B. (2013). A method to differentiate the causes of stiff-knee gait in stroke patients. Gait Posture.

[47] Anderson F.C., Goldberg S.R., Pandy M.G., Delp S.L. (2004). Contributions of muscle forces and toe-off kinematics to peak knee flexion during the swing phase of normal gait: An induced position analysis. J. Biomech..

[48] Chen G., Patten C., Kothari D.H., Zajac F.E. (2005). Gait differences between individuals with post-stroke hemiparesis and non-disabled controls at matched speeds. Gait Posture.

[49] Kobayashi T., Orendurff M.S., Singer M.L., Gao F., Hunt G., Foreman K.B. (2018). Effect of plantarflexion resistance of an ankle-foot orthosis on ankle and knee joint power during gait in individuals post-stroke. J. Biomech..

[50] Tsuchiyama K., Mukaino M., Ohtsuka K., Matsuda F., Tanikawa H., Yamada J., Pongpipatpaiboon K., Kanada Y., Saitoh E., Otaka Y. (2021). Effects of ankle-foot orthoses on the stability of post-stroke hemiparetic gait. Eur. J. Phys. Rehabil. Med..

